# Untargeted metabolomics reveals enhanced antioxidant capacity and key bioactive components of Gougunao No. 2 black tea

**DOI:** 10.3389/fnut.2026.1769834

**Published:** 2026-02-13

**Authors:** Junjie Lu, Jiahao Ai, Cong Wang, Junfeng Jiang, Xinjie Zhou, Yongfa Guo, Zhuangzi Zhong, Jie Zhou, Yang Wu, Ding-kun Liu, Huimin Sun

**Affiliations:** 1 School of Life Sciences, Jinggangshan University, Ji’an, China; 2 Key Laboratory of Jiangxi Province for Biological Invasion and Biosecurity, School of Life Sciences, Jinggangshan University, Ji’an, China; 3 Key Laboratory of Jiangxi Province for Functional Biology and Pollution Control in Red Soil Regions, School of Life Sciences, Jinggangshan University, Ji’an, China

**Keywords:** antioxidant activity, black tea, flavonoids, Gougunao No. 2, untargeted metabolomics

## Abstract

**Introduction:**

Cultivar background may influence antioxidant-related non-volatile metabolite composition of black tea even under identical manufacturing conditions, but the compositional basis and its relationship to assay-specific antioxidant readouts remain unclear.

**Methods:**

We conducted a controlled comparison of black teas produced from Gougunao No. 2 (G2R) and the traditional Gougunao group small-leaf cultivar (BDZ) using the same processing protocol. Untargeted metabolomics was performed using ultra-high-performance liquid chromatography coupled to high-resolution mass spectrometry (UPLC-HRMS) in positive and negative ion modes, followed by differential-metabolite analysis and Kyoto Encyclopedia of Genes and Genomes (KEGG) pathway enrichment based on the differential-metabolite list. Antioxidant-related properties were evaluated using ferric-reducing antioxidant power (FRAP), hydroxyl-radical (·OH) scavenging, ABTS radical-cation (ABTS^•+^) scavenging, and DPPH radical (DPPH•) scavenging, together with total phenolic content and total flavonoid content.

**Results:**

Multivariate analyses showed clear cultivar-dependent separation of global metabolite fingerprints. Positive-ion data highlighted enrichment of amino-acid-related pathways, consistent with higher abundance of 10 annotated amino acids in G2R, whereas negative-ion data emphasized secondary-metabolism pathways centered on flavonoid and phenylpropanoid biosynthesis, accompanied by cultivar-dependent phenolic/flavonoid-related metabolic features. Functionally, G2R exhibited higher FRAP and stronger ·OH scavenging activity than BDZ, together with 10–30% higher total phenolics and total flavonoids, while ABTS^•+^ showed only a small, non-significant difference and DPPH• activities were comparable between cultivars.

**Conclusion:**

Under the tested conditions, cultivar background is associated with coordinated amino-acid- and phenolic/flavonoid-related metabolic differences and with assay-specific antioxidant indices, suggesting that integrated metabolomics-antioxidant profiling may be useful for quality-oriented cultivar evaluation within the scope of this two-cultivar study.

## 1 Introduction

Antioxidant properties of foods and beverages are commonly evaluated using *in vitro* assays based on electron-transfer (ET) and hydrogen-atom-transfer (HAT) principles (1). Because different assays employ distinct radicals and reaction conditions, robust characterization typically requires complementary methods rather than reliance on a single index (2). In tea research, such assay frameworks are frequently used to evaluate how extraction and processing variables influence the yield of phenolics and the resulting antioxidant performance (3).

Tea (*Camellia sinensis*) is consumed worldwide and serves as a major dietary source of redox-active molecules. Even so, antioxidant capacity can differ sharply across tea products, reflecting differences in raw material and processing routes (4). Comparable *in vitro* assay panels are also routinely used for other chemically complex, fermented foods—such as functional yogurts—where they help benchmark how manufacturing steps or formulation choices shift antioxidant behavior (5). Consistent with this, broad surveys of tea chemistry and bioactivity emphasize that health-related effects are not driven by a single constituent, but instead arise from multiple components acting through distinct molecular mechanisms (6). One practical implication is that phenolic composition varies widely among tea types, offering a compositional rationale for their differentiated antioxidant profiles (7).

At the molecular scale, structure-activity analyses point to clear motifs: hydroxylation patterns, and especially catechol or gallate functionalities, tend to strengthen radical scavenging and metal-chelating performance (8). Empirically, links among total phenolics, antioxidant indices, and metal contents have been reported for black and fruit teas, underscoring how phenolic architecture and redox-active features can shape assay readouts (9).

To capture this chemical diversity more comprehensively, non-targeted HRMS has become a powerful way to profile global metabolite fingerprints and to discriminate tea products with distinct chemical characteristics (10). For example, high-throughput UPLC-TOF-MS workflows have been applied to evaluate green tea quality attributes and can detect relatively subtle compositional differences (11). More broadly, metabolomics studies that track time-resolved trajectories provide a useful lens for interpreting condition-dependent metabolic shifts and for relating compositional changes to functional outcomes (12).

Tea metabolite composition is shaped by geographic and climatic factors, underscoring the need to distinguish genotype-driven differentiation from environment-driven variation (13). In chemometrics and metabolomics, orthogonal projections to latent structures (O-PLS) provide an established approach to separate predictive and orthogonal variation and to strengthen interpretability of multivariate models (14). In tea quality research, coupling MS-based identification with sensory evaluation has enabled linking specific metabolites to perception-relevant chemistry, such as astringent contributors in black tea (15).

Tea plant cultivar is a core determinant of metabolite accumulation and quality traits, and integrated transcriptomic-metabolomic analyses have revealed cultivar-linked regulation of metabolic pathways in tea tissues (16). Agronomic and physiological factors can also shift precursor pools, including tea polyphenols and caffeine, thereby influencing downstream product chemistry (17). Within the Gougunao germplasm context, bioactive components such as pectic polysaccharides have been characterized, suggesting distinctive biochemical traits within this cultivar system (18).

In black tea manufacture, non-targeted metabolomics has shown that variation in oxidation/fermentation degree is associated with systematic shifts in non-volatile metabolite profiles (19). Prior studies also report that amino acids, catechins, alkaloids and gallic-acid-related compounds undergo substantial transformations over the course of processing, reflecting coordinated biochemical conversions during manufacture (20). Importantly, because our study applies an identical processing protocol to both materials, we focus on cultivar background as the primary source of compositional divergence—i.e., differences in precursor pools and intrinsic leaf biochemistry that can bias the distribution of downstream metabolites under the same processing conditions. Functional components (e.g., polysaccharides in selenium-enriched tea) have likewise been linked to antioxidant activity, reinforcing the relevance of cultivar-dependent compositional differences for antioxidant phenotypes (21).

Infusion and storage conditions can measurably influence the apparent antioxidant capacity of black tea, and these shifts often track with changes in phenolic abundance (22). Beyond such handling effects, metabolomics has proven useful in practice for tea authentication and quality control: widely targeted workflows can distinguish premium black teas from counterfeit products (23), and both targeted and untargeted strategies have identified metabolites associated with top-grade teas, supporting marker-based evaluation in commercial contexts (24). This applied value aligns with broader syntheses showing that metabolomics is increasingly used in tea research to connect quality evaluation with processing optimization and chemical interpretation (25). At the same time, antioxidant research in oxidative stress and senescence helps explain why redox-active dietary constituents remain of continuing interest for health-related studies (26). Consistent with a multifactorial view of tea antioxidant behavior, mineral element composition has also been reported to covary with antioxidant activity across black teas differing in origin and fermentation conditions (27).

Nevertheless, for black teas manufactured from the newly bred G2R compared with the traditional Gougunao population-type cultivar (BDZ), the degree and mechanistic basis of global metabolic divergence remain insufficiently resolved. It remains unclear which pathways and compound classes underpin cultivar differentiation and whether compositional features translate into consistent functional antioxidant advantages across radical systems.

To address these questions, this study combined UPLC-HRMS-based untargeted metabolomics with multiple *in vitro* antioxidant assays to characterize cultivar-dependent metabolic divergence, identify key pathways contributing to chemical differentiation, and link metabolite enrichment patterns to radical-specific antioxidant mechanisms. The results provide a biochemical foundation for quality evaluation and support high-value utilization of G2R as a functionally differentiated black tea cultivar.

## 2 Materials and methods

### 2.1 Sample collection and processing

In mid-April 2025, fresh tea shoots (one bud with two leaves) of G2R and the BDZ were plucked from commercial tea plantations in Suichuan County, Ji’an City, Jiangxi Province, China. The harvested material was processed into black tea following the local standard DB36/T 1524, “Technical Specifications for Gougunao Black Tea Processing.”

For antioxidant activity assessment, the processed black tea was ground into a uniform fine powder. Exactly 2.00 g of the powder was weighed into a 250 mL Erlenmeyer flask and mixed with 100 mL ultrapure water. The suspension was heated in a 90 °C water bath for 30 min and gently stirred by hand at 10 min intervals to enhance extraction of water-soluble components. After heating, the extract was allowed to reach thermal equilibrium with the environment prior to filtration through a 20 mesh sieve (or equivalent filtration medium). The filtrate was adjusted to 100 mL with ultrapure water, standardized to ensure volume consistency, stored at 4 °C, and subjected to antioxidant activity analysis within 24 h.

### 2.2 Main instruments

Untargeted metabolomic profiling was conducted using a Vanquish Flex UHPLC system hyphenated to an Orbitrap Exploris 120 high-resolution mass spectrometer (Thermo Fisher Scientific, Waltham, MA, United States), fitted with an ACQUITY UPLC HSS T3 column (100 Å, 1.8 μm, 2.1 × 100 mm; Waters, Milford, MA, United States). Sample preparation involved a 5430R refrigerated centrifuge (Eppendorf, Hamburg, Germany), while *in vitro* antioxidant assays were read on a Multiskan FC microplate reader (Thermo Fisher Scientific, Waltham, MA, United States).

### 2.3 Reagents and standards

Unless otherwise stated, all reagents used in this study were sourced at analytical grade purity. Rutin (purity ≥98%) served as the reference compound for total flavonoid determination. Sodium hydroxide (≥96%), ethanol (≥99.7%), aluminum nitrate (≥99%) and sodium nitrite (≥99%) were obtained from commercial sources and used in the colorimetric assay for total flavonoids.

For the ferric-reducing antioxidant power (FRAP) measurements, 2,4,6-tri(2-pyridyl)-s-triazine (TPTZ; ≥99%) and ferrous sulfate heptahydrate (FeSO₄·7H_2_O; ≥99%) were employed. Salicylic acid (≥99.5%) together with hydrogen peroxide solution (about 3% w/w) was used in the hydroxyl radical (·OH) scavenging assay. Folin-Ciocalteu phenol reagent, gallic acid (≥99%) and anhydrous sodium carbonate (≥99.85%) were used for quantifying total phenolic content.

2,2-Diphenyl-1-picrylhydrazyl (DPPH; ≥98%), potassium persulfate (≥99.5%) and 2,2′-azino-bis(3-ethylbenzothiazoline-6-sulphonic acid) (ABTS; ≥98%) were employed in the DPPH and ABTS radical scavenging assays.

### 2.4 Sample preparation for untargeted metabolomics

A 2.0 mL portion was transferred to a 15 mL centrifuge tube, rapidly immersed in liquid nitrogen for snap-freezing, and subsequently subjected to lyophilization. The dried sample was subjected to solvent extraction using 1.0 mL ice-cold methanol/acetonitrile (1:1, v/v) via 30 s of vortex mixing, followed by 30 min of precipitation at −20 °C. The samples were then centrifuged at 12,000 rpm for 10 min at 4 °C.

Next, 850 μL of the clarified supernatant was carefully aliquoted into a sterile, disposable centrifuge tube, and subjected to complete vacuum desiccation. The residue was re-dissolved by adding 150 μL of 50% (v/v) methanol spiked with 5 ppm 2-chlorophenylalanine (IS) and thoroughly vortexed for 30 s. Following a secondary centrifugation at 12,000 rpm for 10 min at 4 °C, the clarified supernatant was filtered through a 0.22 μm pore-size membrane and transferred to LC-MS sample vials. A quality control (QC) pool was generated by collecting 10–20 μL of each filtered extract and homogenizing thoroughly.

### 2.5 Metabolite detection

Metabolite chromatographic separation was carried out using an ACQUITY UPLC HSS T3 column (100 Å, 1.8 μm, 2.1 × 100 mm) with a flow rate of 0.40 mL/min. The column oven was maintained at 40 °C, the autosampler at 8 °C, and a 2 μL injection volume was applied for each sample. Mobile phase A was water with 0.1% formic acid (v/v), while mobile phase B was acetonitrile with 0.1% formic acid (v/v). The gradient elution profile was programmed as follows (Table 1).

**Table 1:** UPLC elution gradient program for positive and negative ion modes.

Time (min)	Mobile phase B (%)
0	5%
1	5%
4.7	95%
6	95%
6.1	5%
8.5	5%

### 2.6 Radical scavenging assays

The radical-scavenging capacity of the black tea extracts was assessed by ABTS, DPPH and hydroxyl radical (·OH) assays, which are commonly applied to evaluate antioxidant activity in tea and other plant-derived foods (28). For each sample, triplicate assays were conducted, and the radical scavenging percentage was determined using the following calculation (Equation 1):


$$\text{Scavenging rate}(\%)=[1-\frac{A_{\text{sample}}-A_{\text{blank}}}{A_{\text{control}}}\;]\times 100\%$$ (1)

Where *A*_control_ is the absorbance of the radical working solution mixed with solvent (i.e., extract replaced by solvent), *A*_sample_ is the absorbance after the radical working solution is reacted with the tea extract, and *A*_blank_ represents the background absorbance of the tea extract measured in the absence of the radical solution.

#### 2.6.1 ABTS assay

In the ABTS assay, the scavenging capacity was evaluated by monitoring the quenching of pre-formed ABTS^•+^ radicals, which was quantified as the decline in absorbance at 734 nm. The assay followed Re et al. (29) with minor modifications. In brief, 180 μL of ABTS^•+^ working solution was dispensed into each well of a 96-well plate and mixed with 20 μL of black tea extract at the indicated concentrations. After incubation for 7 min at room temperature in the dark, absorbance at 734 nm was measured and denoted as *A*_sample_. For the control, tea extract was replaced with anhydrous ethanol (total volume 200 μL; *A*_control_). To correct for sample background, 20 μL of extract was mixed with 180 μL anhydrous ethanol (*A*_blank_). ABTS scavenging (%) was then calculated using the equation given above.

#### 2.6.2 DPPH assay

DPPH scavenging activity was determined based on the color fading of DPPH (deep purple to yellow), quantified by the reduction in absorbance at 517 nm. The procedure was adapted from Nanjo et al. (30). Briefly, 190 μL of DPPH working solution was combined with 10 μL of black tea extract (or Trolox standards at predetermined concentrations) in a 96-well plate. Plates were protected from light with aluminum foil and incubated at room temperature for 30 min. Absorbance at 517 nm was measured as *A*_sample_. The control consisted of solvent replacing the extract (anhydrous ethanol, total 200 μL; *A*_control_), and the sample background was prepared by mixing 10 μL extract with 190 μL anhydrous ethanol (*A*_blank_). The DPPH scavenging (%) was calculated using the same equation as above.

#### 2.6.3 Hydroxyl radical (·OH) scavenging assay

Hydroxyl radical scavenging capacity was assessed using the salicylic acid method (31). In this system, ·OH reacts with salicylic acid to generate a colored product with a maximum absorbance at 510 nm; antioxidants compete with salicylic acid for ·OH, thereby lowering color formation and absorbance. With minor adjustments, 200 μL of 9 mmol/L FeSO₄, 200 μL of 9 mmol/L ethanol-salicylic acid solution, 500 μL of tea extract, and 200 μL of 8.8 mmol/L H_2_O_2_ were added sequentially into a microcentrifuge tube. After vortexing, the mixture was incubated at 37 °C for 30 min. An aliquot (200 μL) was then transferred to a 96-well plate, and absorbance was read at 510 nm. The ·OH scavenging (%) was calculated using the formula described above.

### 2.7 Determination of total phenolic content, total flavonoid content, and total antioxidant capacity

Total phenolic content (TPC), total flavonoid content (TFC), and total antioxidant capacity (TAC) of black tea extracts were determined by spectrophotometric assays with minor modifications (32). TPC was measured using the Folin-Ciocalteu method, TFC by an aluminum nitrate colorimetric assay, and TAC by the ferric-reducing antioxidant power (FRAP) assay. These measurements were used as proxy indicators of phenolic abundance and overall reducing capacity in tea extracts.

#### 2.7.1 TPC (Folin-Ciocalteu)

The TPC was quantified following the Folin-Ciocalteu procedure (33). Diluted tea extracts or gallic acid standards were mixed with Folin reagent working solution and vortexed. After 4 min of incubation in the dark, 2.0 mL of saturated Na_2_CO_3_ solution (75 g/L) was added. The mixture was then kept at room temperature (20–25 °C) in the dark to allow color development. An aliquot (200 μL) was transferred to a 96-well plate, and absorbance at 765 nm was recorded using a microplate reader. TPC was calculated from a gallic acid calibration curve and expressed as mg gallic acid equivalents per gram dry weight (mg GAE/g DW).

#### 2.7.2 TFC (aluminum nitrate method)

The TFC was determined using an aluminum nitrate-based colorimetric method (34). Briefly, 0.5 mL of tea extract was mixed sequentially with 0.3 mL of 5% (w/v) NaNO_2_ and 0.3 mL of 10% (w/v) Al(NO_3_)_3_. After 6 min, 2.0 mL of 1 mol/L NaOH was added, and the final volume was adjusted to 4.5 mL with distilled water. A 200 μL portion was loaded into a 96-well plate, and absorbance was measured at 510 nm. TFC was obtained from a rutin calibration curve and reported as mg rutin equivalents per gram dry weight (mg RE/g DW).

#### 2.7.3 TAC (FRAP)

The TAC was assessed using the FRAP assay, which reflects reducing capacity via the reduction of the Fe^3+^-TPTZ complex to the blue Fe^2+^-TPTZ form, monitored at 593 nm. In each well of a 96-well plate, 190 μL of freshly prepared FRAP reagent was mixed with 10 μL of tea extract or FeSO₄ standard solution. After incubation at 37 °C for 20 min in the dark, absorbance at 593 nm was measured. TAC was calculated from an Fe^2+^ standard curve and expressed as μmol Fe^2+^ equivalents per gram dry weight (μmol Fe^2+^/g DW). All TPC, TFC, and FRAP measurements were performed in triplicate.

### 2.8 Statistical analysis

Raw UPLC-HRMS data were processed in the instrument vendor’s software to export a peak-intensity table. Baseline correction, peak alignment, and signal normalization were then applied following routine untargeted metabolomics workflows. The resulting matrix was log10-transformed and Pareto-scaled before multivariate analysis. PCA (unsupervised) and OPLS-DA (supervised) were performed in SIMCA (v14.1; Umetrics, Umeå, Sweden). Model performance was summarized by *R*^2^ and *Q*^2^, and potential overfitting was checked using 200 permutation tests; models with a *Q*^2^ intercept <0.05 were considered acceptable under commonly used OPLS-DA validation criteria.

Differential metabolites between G2R and the traditional Gougunao small-leaf cultivar (BDZ) were identified by combining OPLS-DA variable importance in projection (VIP) scores with univariate testing. Features meeting VIP > 1.0 and Student’s *t*-test *p* < 0.05 were treated as significantly different. Fold change (FC) was calculated as the mean abundance in G2R divided by the mean abundance in BDZ. KEGG pathway enrichment was conducted in MetaboAnalyst (v5.0) using the differential-metabolite list, and pathways with adjusted *p* < 0.05 were regarded as significantly enriched, consistent with prior reports (35, 36).

For antioxidant-related indices (ABTS, DPPH, ·OH scavenging activity, TPC, TFC, and FRAP), values are reported as mean ± SD from triplicate measurements (*n* = 3). Differences between the two cultivars were evaluated using two-tailed Student’s *t*-tests in IBM SPSS Statistics (v26.0; IBM Corp., Armonk, NY, United States), with *p* < 0.05 considered significant and *p* < 0.01 considered highly significant. Figures were generated in OriginPro (OriginLab Corp., Northampton, MA, United States).

## 3 Results

### 3.1 Quality assessment of UPLC-HRMS data

**Figure 1:**
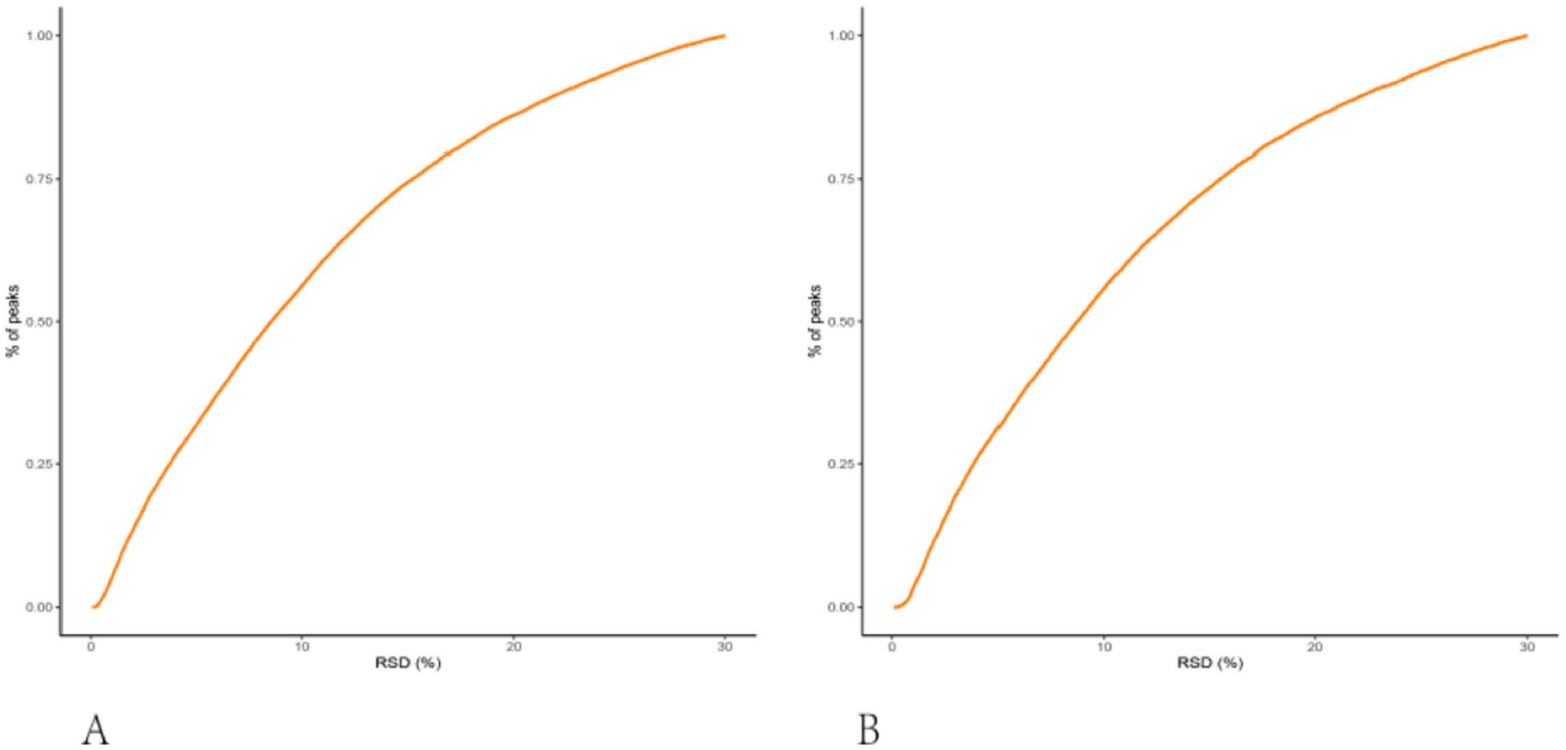
Scatter plots of QC injections from the LC-MS metabolomics workflow, used to assess analytical stability and repeatability. **(A)** Positive-ion mode; **(B)** negative-ion mode. Each point represents one QC run (pooled QC sample), and the tight clustering indicates stable instrument performance across the analytical batch.

To monitor the stability and reproducibility of the UPLC-HRMS platform, pooled QC samples were periodically analyzed across the entire run in both positive and negative ion modes. Inspection of QC clustering in the multivariate score plots, together with the relative standard deviations (RSDs) of QC peak intensities, showed that over 65% of detected features exhibited RSDs below 30% in both modes (Figure 1), demonstrating satisfactory analytical repeatability.

### 3.2 Principal component analysis of metabolic profiles

**Figure 2:**
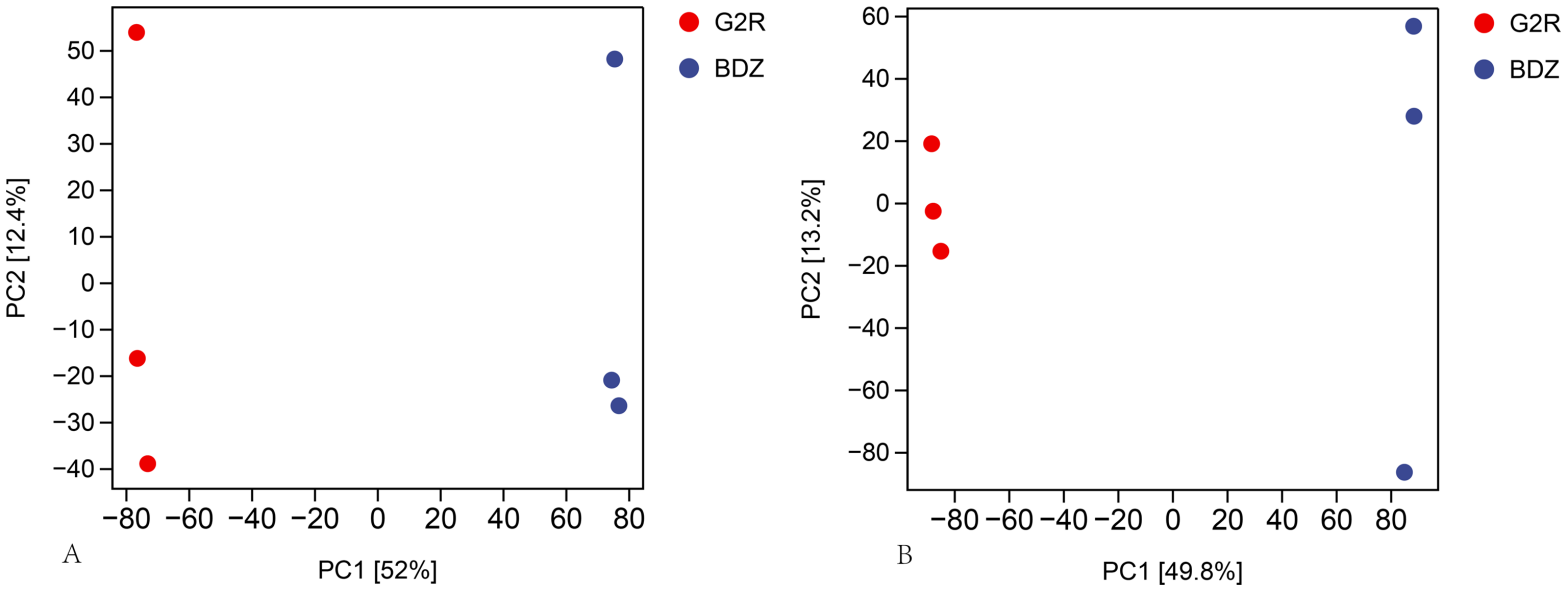
Principal component analysis (PCA) score plots of sample-level non-volatile metabolite profiles for G2R and the BDZ. **(A)** Positive-ion mode; **(B)** negative-ion mode. Each point represents one biological replicate. Red points denote G2R and blue points denote BDZ. PC1 and PC2 explain 52.0 and 12.4% of the variance in positive-ion mode and 49.8 and 13.2% of the variance in negative-ion mode, respectively, indicating clear cultivar-dependent separation.

Principal component analysis (PCA) was applied to the untargeted metabolomic profiles of the finished black teas from G2R and the traditional Gougunao group small-leaf cultivar. The first two principal components (PC1 and PC2) explained 52.0 and 12.4% of the variability in positive ion mode, compared to 49.8 and 13.2% in negative ion mode. As illustrated in the two-dimensional PCA score plots (Figure 2), the two cultivars were distinctly separated along the PC1 axis in both ion modes, and biological replicates within each group clustered tightly. This pattern reflects stable, cultivar-dependent separation at the level of overall metabolite composition between G2R and the traditional Gougunao group small-leaf black tea.

### 3.3 Orthogonal partial least squares discriminant analysis

**Figure 3:**
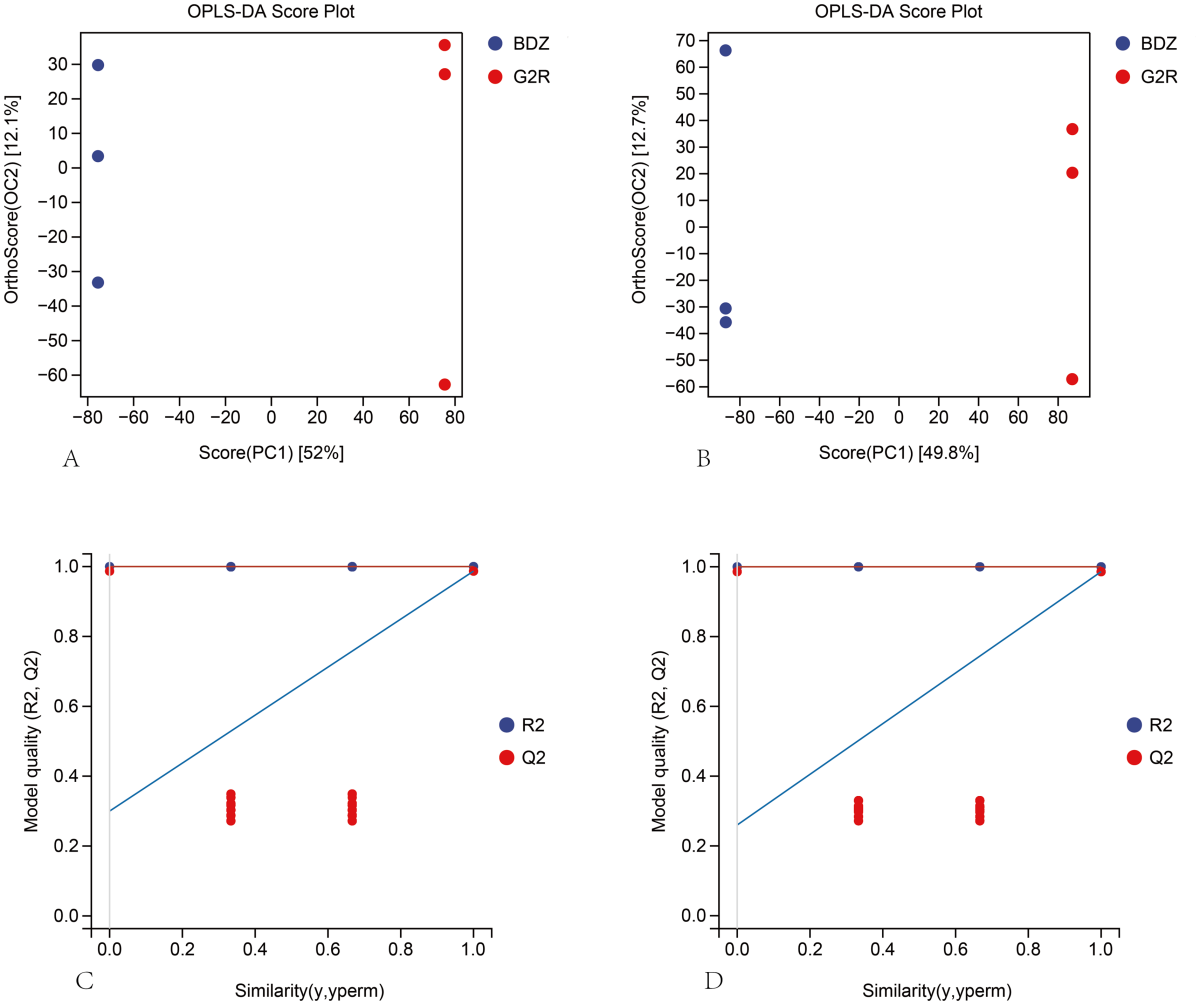
OPLS-DA score plots and permutation-test validation of metabolite profiles for G2R and the BDZ. **(A)** OPLS-DA score plot in positive-ion mode; **(B)** OPLS-DA score plot in negative-ion mode. Each point represents one biological replicate; red points denote G2R and blue points denote BDZ. The separation between cultivars along the predictive component indicates cultivar-dependent differences in global metabolite profiles. **(C)** 200-permutation test for the positive-ion OPLS-DA model; **(D)** 200-permutation test for the negative-ion OPLS-DA model. In the permutation plots, *R*^2^ is shown in blue and *Q*^2^ in red; the permuted *R*^2^/*Q*^2^ values are lower than those of the original model, and the *Q*^2^ intercepts are below 0.05, indicating acceptable model robustness and limited risk of overfitting.

To further characterize cultivar-dependent metabolic differences, a supervised multivariate discriminant analysis (OPLS-DA) was applied to the untargeted metabolomics dataset. In the OPLS-DA score plots (Figures 3A,B), G2R and the traditional Gougunao group small-leaf black tea formed two well-resolved clusters along the predictive component (PC1) in both positive (Figure 3A) and negative (Figure 3B) ion modes, with biological replicates for each cultivar grouping closely together. This clear separation corroborates the existence of marked and reproducible differences in global metabolite composition between the two black teas.

Consistent with the subsequent detection of differential metabolites, the positive-ion OPLS-DA model was predominantly influenced by variables annotated as amino acids and their derivatives, whereas the negative-ion model captured a larger proportion of variance linked to flavonoids and phenolic acids. This pattern points to multidimensional divergence between the cultivars in both flavor associated and bioactive metabolite pools.

Model reliability was further examined using 200-permutation tests. As shown in Figures 3C,D, the permutation-derived *R*^2^ (blue) and *Q*^2^ (red) values for both ion modes were all markedly lower than those of the original models, and the *Q*^2^ regression line intercepts were less than 0.05. These results indicate that the OPLS-DA models were not over-fitted and that the observed discrimination between cultivars reflects true underlying metabolic differences rather than random noise.

### 3.4 KEGG pathway enrichment of differential metabolites

**Figure 4:**
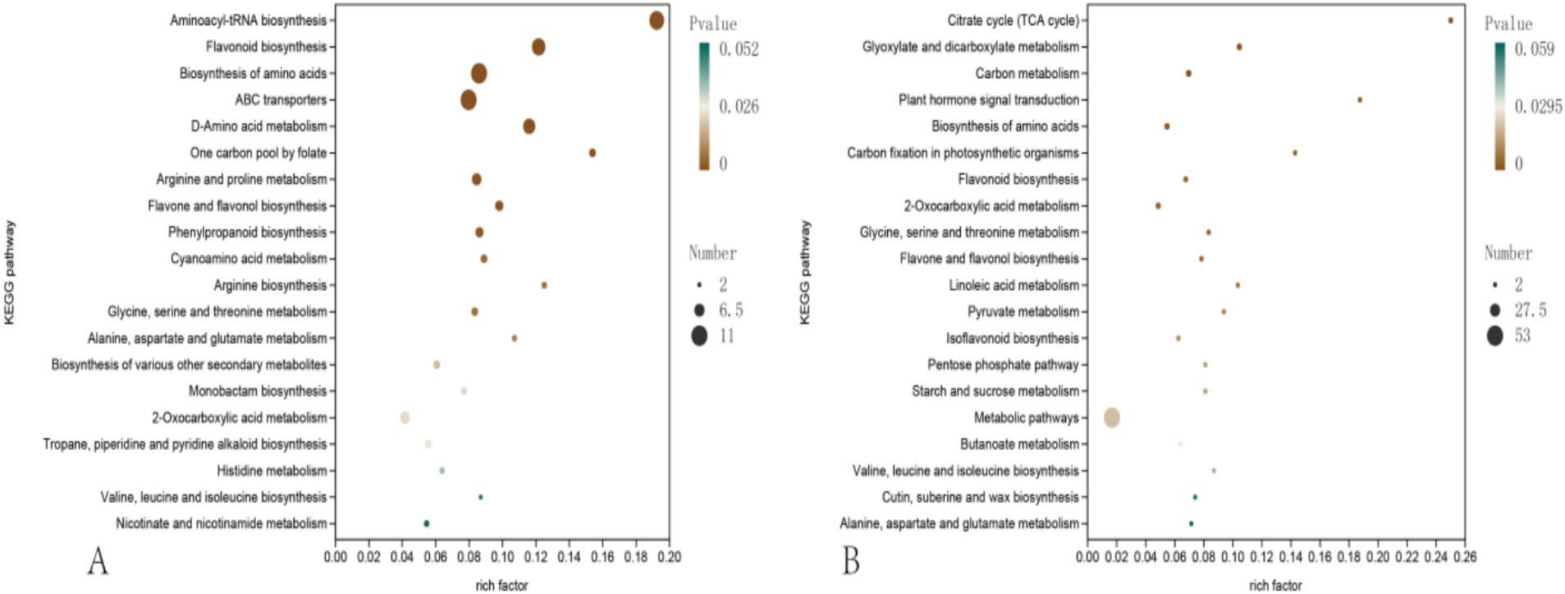
KEGG pathway enrichment bubble plots based on differential metabolites between G2R and the BDZ. **(A)** Positive-ion mode; **(B)** negative-ion mode. Each bubble represents one enriched KEGG pathway. The *x*-axis shows the rich factor (ratio of differential metabolites mapped to a pathway to all annotated metabolites in that pathway); bubble size indicates the number of differential metabolites mapped to the pathway; bubble color reflects the adjusted *p*-value. Here, “pathway analysis” denotes KEGG pathway enrichment performed on the differential-metabolite list. Panels **(A,B)** correspond to ionization modes rather than cultivars.

For clarity, we performed KEGG pathway enrichment analysis (pathway-level enrichment) based on the differential-metabolite list, and the enriched pathways are summarized in Figure 4. Under positive-ion acquisition, the enriched terms clustered mainly around amino-acid metabolism. The top pathways included aminoacyl-tRNA biosynthesis and amino-acid biosynthesis, together with several amino-acid-specific routes (e.g., glycine/serine/threonine metabolism and arginine biosynthesis). Notably, consistent with the enrichment of amino-acid-related pathways, 10 annotated amino acids [e.g., L-proline and L-(+)-aspartic acid] showed higher abundance in G2R (Figure 4 and Supplementary Table S1), suggesting a cultivar-associated difference in amino-acid pools that may influence taste-related precursors and downstream metabolism.

In contrast, negative-ion data emphasized secondary-metabolism pathways, with flavonoid biosynthesis and phenylpropanoid biosynthesis standing out. In agreement with these enrichments, several annotated flavonoid-related metabolites (e.g., quercetin- and rutin-related flavonoid features) were identified among the differential metabolites (Supplementary Table S2), consistent with the pathway-level enrichment results (Figure 4). Overall, the negative-ion dataset indicates cultivar-dependent differences in phenolic/flavonoid-related metabolic features under identical processing conditions, as supported by pathway-level enrichment (Figure 4) and metabolite-level annotations (Supplementary Table S2).

### 3.5 Antioxidant activity analysis

To enable reliable quantification of antioxidant activity and related constituents in the black tea extracts, standard curves were generated for six widely used assays: ABTS, DPPH, hydroxyl radical (·OH) scavenging, FRAP, TPC and TFC. Trolox, Fe^2+^, gallic acid and rutin served as the respective reference standards for these measurements. As shown in Table 2, all calibration curves displayed excellent linearity over the tested concentration ranges, with coefficients of determination (*R*^2^) exceeding 0.99, confirming that these assays were well suited for quantitative assessment of the antioxidant characteristics of the samples.

**Table 2:** Parameters of the calibration curves for the antioxidant assays.

Assay	Parameter	Standard	Calibration curve	Linear range	*R* ^2^
ABTS assay	Radical scavenging	Trolox	*y* = −0.0057*x* + 0.6986	0–100 nmol/L	0.9959
DPPH assay	Radical scavenging	Trolox	*y* = −0.0053*x* + 0.652	0–100 nmol/L	0.9925
Hydroxyl radical	Radical scavenging	Trolox	*y* = −0.0042*x* + 0.5001	0–100 μmol/L	0.9915
FRAP assay	Antioxidant capacity	Fe^2+^	*y* = 0.9919*x* − 0.0315	0–1.0 nmol/L	0.9906
Folin-Ciocalteu	Total phenolics	Gallic acid	*y* = 0.0057*x* + 0.0418	0–100 μg/mL	0.9991
Al(NO_3_)_3_ colorimetric	Total flavonoids	Rutin	*y* = 0.0118*x* + 0.0195	0–10 μg/mL	0.9939

### 3.6 Free radical scavenging activities

**Figure 5:**
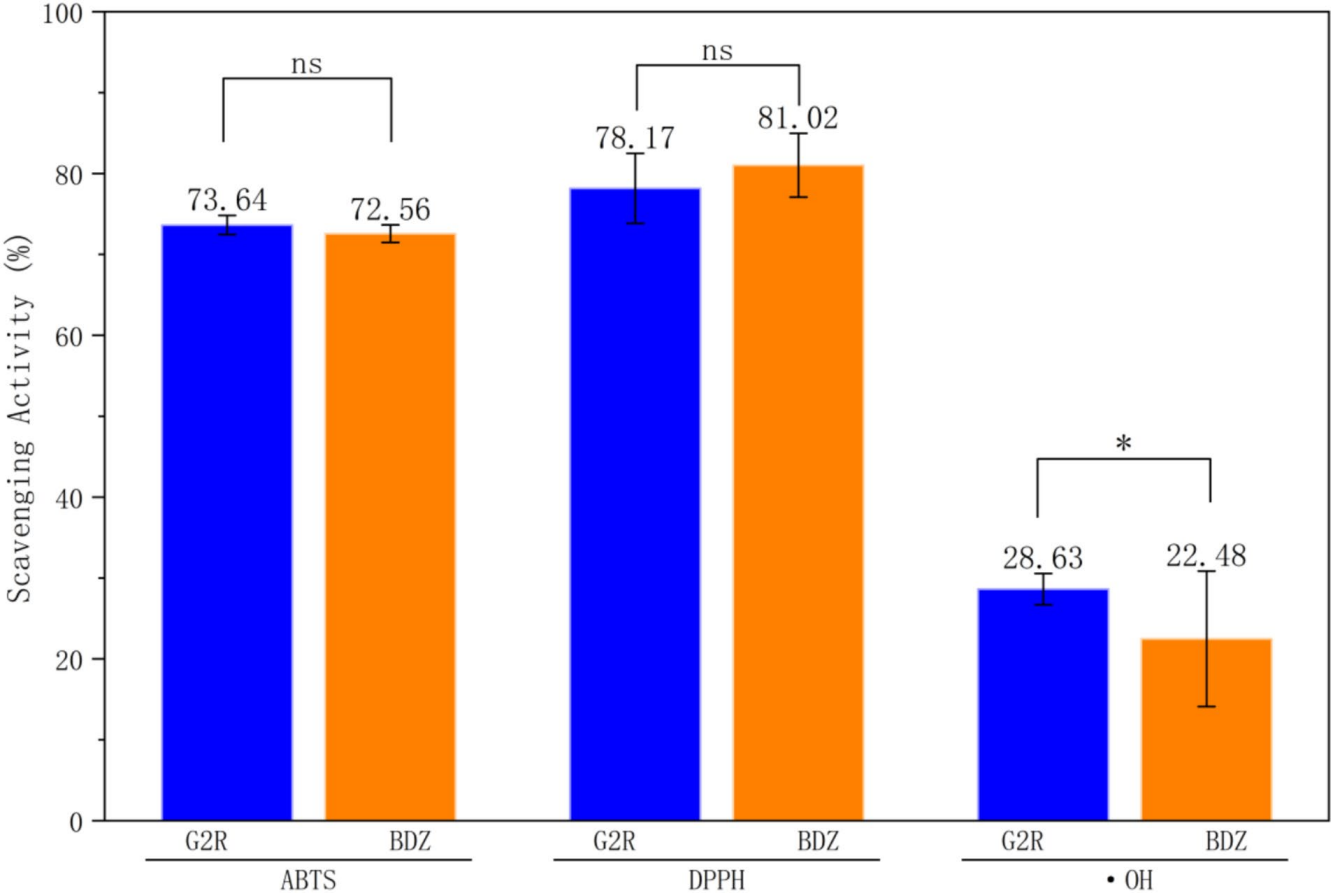
Comparison of free radical scavenging activities (ABTS^•+^, •OH, and DPPH^•^) of black tea extracts from G2R and the BDZ. Data are presented as mean ± SD (*n* = 3). ns, not significant (*p* > 0.05); *p < 0.05.

As illustrated in Figure 5, the ABTS^•+^, DPPH• and ·OH assays showed assay-dependent variation in antioxidant performance between the two black teas. In the aqueous ABTS^•+^ system, G2R displayed a marginally higher radical scavenging rate (73.64 ± 0.88%) than the traditional Gougunao group small-leaf tea (BDZ, 72.59 ± 0.79%), but this difference was not statistically significant (*p* > 0.05).

In contrast, a clear distinction emerged in the ·OH scavenging assay: G2R achieved a significantly higher hydroxyl radical scavenging rate than BDZ (28.63 ± 1.15% vs. 22.48 ± 3.21%, *p* < 0.05), representing an increase of roughly 27% relative to BDZ and indicating a significantly higher ability of G2R to quench hydroxyl radicals under the tested conditions. In the lipophilic DPPH• assay, BDZ exhibited a slightly higher scavenging rate (81.02 ± 3.19%) compared with G2R (78.17 ± 3.38%), but the difference was small (about 3 percentage points) and did not reach statistical significance (*p* > 0.05).

### 3.7 Total phenolic content, total flavonoid content, and total antioxidant capacity

As shown in Table 3, G2R exhibited stronger *in vitro* antioxidant performance than the traditional Gougunao small-leaf tea (BDZ), consistent with its higher abundance of antioxidant-associated metabolites. The TPC of G2R reached 62.00 ± 0.82 mg GAE/g, exceeding BDZ (56.28 ± 1.62 mg GAE/g) by 10.2% (*p* < 0.05).

**Table 3:** Contents of total phenolics, total flavonoids and total antioxidant capacity (FRAP) in black tea extracts from G2R and the BDZ.

Indicator	G2R	BDZ	Change (%)	*p-value
Total phenolic content	62.00 ± 0.82 mg GAE/g	56.28 ± 1.62 mg GAE/g	+10.2%	*p < 0.05
Total flavonoid content	10.20 ± 1.33 mg RE/g	8.82 ± 0.62 mg RE/g	+15.6%	**p < 0.01
Total antioxidant capacity	523.4 ± 24.1 μmol Fe^2+^/g	401.3 ± 18.5 μmol Fe^2+^/g	+30.4%	*p < 0.05

Data are expressed as mean ± SD (*n* = 3). GAE, gallic acid equivalent; RE, Rutin equivalent. *p < 0.05, **p < 0.01.

A similar pattern was observed for TFC. G2R contained 10.20 ± 1.33 mg RE/g, significantly higher than BDZ (8.82 ± 0.62 mg RE/g; *p* < 0.01), corresponding to a 15.6% increase. These compositional differences were reflected in reducing power: the FRAP value of G2R was 523.4 ± 24.1 μmol Fe^2+^/g, which was 30.4% higher than that of BDZ (401.3 ± 18.5 μmol Fe^2+^/g; *p* < 0.05). Overall, the data indicate that G2R shows higher levels of antioxidant-related components and higher FRAP/·OH indices than BDZ in this comparison.

## 4 Discussion

### 4.1 Cultivar-dependent global metabolic divergence between G2R and BDZ

The distinct separation between G2R and the traditional population-type cultivar (BDZ) in the multivariate analyses indicates a pronounced cultivar effect on the global non-volatile metabolite fingerprint under the same processing protocol (Figures 2, 3). Rather than a superficial “pattern shift,” this separation suggests coordinated cultivar-associated differences spanning primary and secondary metabolic features. Consistent with this global divergence, pathway-level enrichment further highlights amino-acid- and phenolic/flavonoid-related signals as major components underlying the cultivar contrast (Figure 4 and Supplementary Tables S1, S2).

### 4.2 Enrichment of amino acid and flavonoid pathways underlying antioxidant potential

The KEGG pathway enrichment provides a pathway-level view of metabolic features differentiating the cultivars (Figure 4). Under positive-ion acquisition, enriched terms clustered mainly around amino-acid metabolism, including aminoacyl-tRNA biosynthesis and biosynthesis of amino acids, together with amino-acid-specific routes such as glycine/serine/threonine metabolism and arginine biosynthesis (Figure 4A). Consistent with these pathway signals, 10 annotated amino acids showed higher abundance in G2R (Supplementary Table S1), supporting a cultivar-associated difference in amino-acid pools under standardized processing conditions.

In contrast, negative-ion data emphasized secondary-metabolism pathways, with flavonoid biosynthesis and phenylpropanoid biosynthesis among the prominent enriched terms (Figure 4B). As Figure 4 summarizes enrichment at the pathway level, metabolite-level annotations supporting these signals are provided in Supplementary Table S2 (e.g., quercetin- and rutin-related flavonoid features, together with other annotated phenolic/flavonoid-related metabolites). It is important to note that enrichment reflects over-representation at the pathway level and may include metabolites showing either higher or lower abundance between cultivars; therefore, we interpret these results as evidence for cultivar-dependent differences in phenolic/flavonoid-related metabolic features rather than asserting changes in individual single metabolites based on the enrichment plot alone.

### 4.3 *In vitro* antioxidant assays reveal assay-specific differences between G2R and BDZ

Because antioxidant assays probe different radical chemistries and reaction environments, cultivar differences are expected to be assay-dependent rather than uniform across all systems. In this study, the clearest differences were observed in ·OH scavenging and ferric-reducing power (FRAP), both of which were higher in G2R, whereas ABTS^•+^ scavenging showed only a small, non-significant difference and DPPH• scavenging was comparable or slightly higher in BDZ (Figure 5 and Table 3). This pattern is chemically plausible because FRAP is dominated by ET capacity, and the ·OH assay (Fe^2+^-H_2_O_2_ system) can be influenced by both direct radical quenching and metal-chelation-related suppression of radical generation. By contrast, ABTS and DPPH measure scavenging of relatively stable radicals and can be influenced by reaction kinetics and matrix/solvent effects; for example, DPPH• is more lipophilic and may be biased by partitioning and accessibility in complex extracts (37). Overall, the assay-specific outcomes indicate that cultivar differences are more evident in aqueous-phase redox/Fenton-related assays than across all radical platforms, consistent with the metabolomics-derived phenolic/flavonoid-related features (Figure 4 and Supplementary Tables S1, S2).

### 4.4 Quantification of functional components corroborates metabolomic findings

The higher TPC, TFC, and FRAP values observed for G2R provide a focused biochemical counterpart to the untargeted metabolomics results. In many tea matrices, variation in phenolic composition tends to parallel changes in common *in vitro* antioxidant indices, which helps contextualize the TPC/TFC-FRAP consistency seen here (38). This association is chemically plausible because phenolics often contribute strongly to ET-based readouts by facilitating electron donation and stabilizing radical intermediates. Accordingly, the elevated TPC/TFC in G2R offers a plausible compositional basis for its higher FRAP and related antioxidant indices, while recognizing that these assays reflect chemical reactivity rather than *in vivo* efficacy.

In addition, the stronger ·OH scavenging observed for G2R is compatible with this compositional pattern. Because the ·OH assay used here is based on an Fe^2+^-H_2_O_2_ (Fenton) system, phenolic/flavonoid constituents may contribute not only via electron donation but also via metal chelation, which can attenuate ·OH generation and/or directly quench radicals. This interpretation is supported by pathway-level enrichment of flavonoid/phenylpropanoid-related pathways (Figure 4) and by the annotated phenolic/flavonoid-related metabolites summarized in Supplementary Table S2.

However, these measurements primarily reflect chemical reactivity under assay conditions rather than biological efficacy. Interpretation should therefore remain cautious, since *in vivo* antioxidant defense depends on absorption, metabolism, and compartment-specific redox control that cannot be captured by simplified chemical assays alone (39).

### 4.5 Limitations and future perspectives

Several limitations should be acknowledged. First, sampling was restricted to a single region and season, and only one standardized processing protocol was applied; terroir and processing parameters are known to reshape tea metabolite profiles and may interact with cultivar effects (40). Second, antioxidant evaluation relied on chemical assays, which are efficient for screening but do not capture the complexity of physiological oxidative stress responses (41).

Future work should include multi-location and multi-season sampling and apply targeted quantification of the metabolite features highlighted in this study (Supplementary Tables S1, S2) to better disentangle genotype- and environment-driven effects. Mechanistically, integrating metabolomics with transcriptomics/proteomics would enable direct testing of regulatory hypotheses (e.g., pathway-enzyme regulation) underlying cultivar-associated differences in amino-acid and phenolic/flavonoid features (42). In addition, cell-based or *in vivo* assays would be valuable to determine whether the observed chemical differences translate into biologically meaningful outcomes under oxidative stress conditions. Finally, coupling chemical characterization with sensory evaluation and consumer studies could connect compositional differences to flavor perception and market acceptance, supporting quality-oriented breeding and product development (43).

## 5 Conclusion

This study aimed to determine whether cultivar background, under an identical black-tea processing protocol, is associated with systematic differences in non-volatile metabolite composition and assay-specific antioxidant behaviors between G2R and the BDZ. By integrating UPLC-HRMS-based untargeted metabolomics (positive/negative ion acquisition), KEGG pathway enrichment, and a panel of *in vitro* antioxidant assays, we sought to (i) characterize cultivar-dependent metabolic divergence, (ii) identify pathway-level features contributing to chemical differentiation, and (iii) relate these compositional features to antioxidant readouts.

Our results showed clear separation between G2R and BDZ in the global metabolite fingerprint, indicating a pronounced cultivar effect under standardized processing conditions. KEGG pathway enrichment indicated that positive-ion features were primarily associated with amino-acid metabolism (e.g., aminoacyl-tRNA biosynthesis and amino-acid biosynthesis) (Figure 4), and 10 annotated amino acids showed higher abundance in G2R (Supplementary Table S1). In contrast, the negative-ion dataset highlighted secondary-metabolism pathways, with flavonoid and phenylpropanoid biosynthesis enriched (Figure 4), supporting cultivar-dependent differences in phenolic/flavonoid-related metabolic features summarized in Supplementary Table S2.

Functionally, G2R exhibited higher FRAP and stronger ·OH scavenging activity than BDZ, together with higher total phenolic and TFCs, whereas ABTS and DPPH activities showed limited or no significant differences. This assay-specific pattern indicates that cultivar differences are more evident in aqueous-phase redox and Fenton-system-related assays than across all radical platforms, consistent with the metabolomics-derived enrichment of phenolic/flavonoid-related features.

Taken together, these findings advance our understanding by providing a controlled, cultivar-focused dataset that links pathway-level metabolomic features to antioxidant assay outcomes under identical processing. Future work should test mechanistic hypotheses suggested here—such as the roles of phenolic substitution patterns (e.g., hydroxylation, glycosylation, and galloylation in general), redox potential, and metal chelation—in driving assay-specific behaviors, ideally using targeted quantification, process-stage sampling, and broader cultivar panels. While the current conclusions are based on *in vitro* chemical assays and two cultivars, the integrated workflow offers a practical framework for quality-oriented evaluation and cultivar selection under standardized manufacturing conditions.

## Data Availability

The raw data supporting the conclusions of this article will be made available by the authors, without undue reservation.
