# Supplementary material for: Untargeted metabolomics reveals enhanced antioxidant capacity and key bioactive components of Gougunao No. 2 black tea
Source: Front Nutr. 2026 Feb 13;13:1769834. doi: 10.3389/fnut.2026.1769834 (PMC12946027; doi:10.3389/fnut.2026.1769834)
Supplement: Supplementary file 1 [file Data_Sheet_1.PDF]

Supplementary Tables

Table S1. Annotated amino-acid-related metabolites/features supporting the positive-ion KEGG enrichment (G2R vs BDZ).

Direction	Metabolite	KEGG compound	Internal ID	PathwayID	Pathway	Level2	Pathway p-value	Pathway FDR	Type
Up in G2R	L-(+)-Aspartic acid	C00049	MP3179.pos	csin00220; csin00250; csin00260; csin00261; csin00270; csin00300; csin00340; csin00410; csin00460; csin00470; csin00630; csin00710; csin00760; csin00770; csin00970; csin00997; csin00998; csin01100; csin01110; csin01200; csin01210; csin01230; csin01240; csin02010	2-Oxocarboxylic acid metabolism; ABC transporters; Alanine, aspartate and glutamate metabolism; Aminoacyl-tRNA biosynthesis; Arginine biosynthesis; Biosynthesis of amino acids; Biosynthesis of cofactors; Biosynthesis of secondary metabolites; Biosynthesis of various antibiotics; Biosynthesis of various other secondary metabolites; Carbon fixation in photosynthetic organisms; Carbon metabolism; Cyanoamino acid metabolism; Cysteine and methionine metabolism; D-Amino acid metabolism; Glycine, serine and threonine metabolism; Glyoxylate and dicarboxylate metabolism; Histidine metabolism; Lysine biosynthesis; Metabolic pathways; Monobactam biosynthesis; Nicotinate and	Amino acid metabolism; Biosynthesis of other secondary metabolites; Carbohydrate metabolism; Energy metabolism; Global and overview maps; Membrane transport; Metabolism of cofactors and vitamins; Metabolism of other amino acids; Translation	1.19116036757139e-08	5.3006636356927e-07	Amino acid (canonical)

					nicotinamide metabolism; Pantothenate and CoA biosynthesis; beta-Alanine metabolism				
Up in G2R	L-(-)-Asparagine	C00152	MP3117.pos	csin00250; csin00460; csin00970; csin01100; csin01110; csin01230	Alanine, aspartate and glutamate metabolism; Aminoacyl-tRNA biosynthesis; Biosynthesis of amino acids; Biosynthesis of secondary metabolites; Cyanoamino acid metabolism; Metabolic pathways	Amino acid metabolism; Global and overview maps; Metabolism of other amino acids; Translation	1.19116036757139e-08	5.3006636356927e-07	Amino acid (canonical)
Up in G2R	L-Histidine	C00135	MP4431.pos	csin00340; csin00410; csin00470; csin00970; csin01100; csin01110; csin01230; csin02010	ABC transporters; Aminoacyl-tRNA biosynthesis; Biosynthesis of amino acids; Biosynthesis of secondary metabolites; D-Amino acid metabolism; Histidine metabolism; Metabolic pathways; beta-Alanine metabolism	Amino acid metabolism; Global and overview maps; Membrane transport; Metabolism of other amino acids; Translation	1.19116036757139e-08	5.3006636356927e-07	Amino acid (canonical)
Up in G2R	L-Isoleucine	C00407	MP3054.pos	csin00280; csin00290; csin00460; csin00960; csin00966; csin00970; csin01100; csin01110; csin01210; csin01230; csin02010	2-Oxocarboxylic acid metabolism; ABC transporters; Aminoacyl-tRNA biosynthesis; Biosynthesis of amino acids; Biosynthesis of secondary metabolites; Cyanoamino acid metabolism; Glucosinolate biosynthesis; Metabolic pathways; Tropane, piperidine and pyridine alkaloid biosynthesis; Valine, leucine and isoleucine	Amino acid metabolism; Biosynthesis of other secondary metabolites; Global and overview maps; Membrane transport; Metabolism of other amino acids; Translation	1.19116036757139e-08	5.3006636356927e-07	Amino acid (canonical)

					biosynthesis; Valine, leucine and isoleucine degradation				
Up in G2R	L-Lysine	C00047	MP3919.pos	csin00300; csin00310; csin00470; csin00780; csin00960; csin00970; csin00997; csin01100; csin01110; csin01210; csin01230; csin02010	2-Oxocarboxylic acid metabolism; ABC transporters; Aminoacyl-tRNA biosynthesis; Biosynthesis of amino acids; Biosynthesis of secondary metabolites; Biosynthesis of various other secondary metabolites; Biotin metabolism; D-Amino acid metabolism; Lysine biosynthesis; Lysine degradation; Metabolic pathways; Tropane, piperidine and pyridine alkaloid biosynthesis	Amino acid metabolism; Biosynthesis of other secondary metabolites; Global and overview maps; Membrane transport; Metabolism of cofactors and vitamins; Metabolism of other amino acids; Translation	1.19116036757139e-08	5.3006636356927e-07	Amino acid (canonical)
Up in G2R	L-Methionine	C00073	MP4111.pos	csin00270; csin00470; csin00670; csin00920; csin00966; csin00970; csin01100; csin01110; csin01210; csin01230; csin01240	2-Oxocarboxylic acid metabolism; Aminoacyl-tRNA biosynthesis; Biosynthesis of amino acids; Biosynthesis of cofactors; Biosynthesis of secondary metabolites; Cysteine and methionine metabolism; D-Amino acid metabolism; Glucosinolate biosynthesis; Metabolic pathways; One carbon pool by folate; Sulfur metabolism	Amino acid metabolism; Biosynthesis of other secondary metabolites; Energy metabolism; Global and overview maps; Metabolism of cofactors and vitamins; Metabolism of other amino acids; Translation	1.19116036757139e-08	5.3006636356927e-07	Amino acid (canonical)
Up in G2R	L-Proline	C00148	MP2198.pos	csin00330; csin00470; csin00970; csin01100; csin01110; csin01230;	ABC transporters; Aminoacyl-tRNA biosynthesis; Arginine and proline metabolism; Biosynthesis of	Amino acid metabolism; Global and overview maps; Membrane transport; Metabolism of other amino acids;	1.19116036757139e-08	5.3006636356927e-07	Amino acid (canonical)

				csin02010	amino acids; Biosynthesis of secondary metabolites; D-Amino acid metabolism; Metabolic pathways	Translation			
Up in G2R	L-Threonine	C00188	MP2393.pos	csin00260; csin00261; csin00290; csin00470; csin00860; csin00970; csin01100; csin01110; csin01230; csin02010	ABC transporters; Aminoacyl-tRNA biosynthesis; Biosynthesis of amino acids; Biosynthesis of secondary metabolites; D-Amino acid metabolism; Glycine, serine and threonine metabolism; Metabolic pathways; Monobactam biosynthesis; Porphyrin metabolism; Valine, leucine and isoleucine biosynthesis	Amino acid metabolism; Biosynthesis of other secondary metabolites; Global and overview maps; Membrane transport; Metabolism of cofactors and vitamins; Metabolism of other amino acids; Translation	1.19116036757139e-08	5.3006636356927e-07	Amino acid (canonical)
Up in G2R	L-Tryptophan	C00078	MN7829.neg	csin00260; csin00380; csin00400; csin00966; csin00970; csin00997; csin01100; csin01110; csin01210; csin01230; csin01240	2-Oxocarboxylic acid metabolism; Aminoacyl-tRNA biosynthesis; Biosynthesis of amino acids; Biosynthesis of cofactors; Biosynthesis of secondary metabolites; Biosynthesis of various other secondary metabolites; Glucosinolate biosynthesis; Glycine, serine and threonine metabolism; Metabolic pathways; Phenylalanine, tyrosine and tryptophan biosynthesis; Tryptophan metabolism	Amino acid metabolism; Biosynthesis of other secondary metabolites; Global and overview maps; Translation	1.19116036757139e-08	5.3006636356927e-07	Amino acid (canonical)

Up in G2R	L-Tyrosine	C00082	MP5852.pos	csin00130; csin00261; csin00350; csin00360; csin00400; csin00460; csin00730; csin00940; csin00950; csin00965; csin00966; csin00970; csin00997; csin00998; csin01100; csin01110; csin01210; csin01230; csin01240	2-Oxocarboxylic acid metabolism; Aminoacyl-tRNA biosynthesis; Betalain biosynthesis; Biosynthesis of amino acids; Biosynthesis of cofactors; Biosynthesis of secondary metabolites; Biosynthesis of various antibiotics; Biosynthesis of various other secondary metabolites; Cyanoamino acid metabolism; Glucosinolate biosynthesis; Isoquinoline alkaloid biosynthesis; Metabolic pathways; Monobactam biosynthesis; Phenylalanine metabolism; Phenylalanine, tyrosine and tryptophan biosynthesis; Phenylpropanoid biosynthesis; Thiamine metabolism; Tyrosine metabolism; Ubiquinone and other terpenoid-quinone biosynthesis	Amino acid metabolism; Biosynthesis of other secondary metabolites; Global and overview maps; Metabolism of cofactors and vitamins; Metabolism of other amino acids; Translation	1.19116036757139e-08	5.3006636356927e-07	Amino acid (canonical)

Table S2. Annotated phenolic/flavonoid-related metabolites/features supporting the negative-ion KEGG enrichment (G2R vs BDZ).

Direction	Metabolite	KEGG compound	Internal ID	PathwayID	Pathway	Pathway p-value	Pathway FDR
Down in G2R	(-)-Epicatechin	C09727	MP11031.pos	csin00941; csin01110	Biosynthesis of secondary metabolites; Flavonoid biosynthesis	1.14816623097032e-08	5.3006636356927e-07
Down in G2R	Epigallocatechin	C12136	MP11945.pos	csin00941; csin01110	Biosynthesis of secondary metabolites; Flavonoid biosynthesis	1.14816623097032e-08	5.3006636356927e-07
Down in G2R	Rutin	C05625	MP25174.pos	csin00944; csin01110	Biosynthesis of secondary metabolites; Flavone and flavonol biosynthesis	9.80739906527833e-06	0.0002182146292024
Up in G2R	Apigenin	C01477	MN11056.neg	csin00941; csin00943; csin00944; csin01110	Biosynthesis of secondary metabolites; Flavone and flavonol biosynthesis; Flavonoid biosynthesis; Isoflavonoid biosynthesis	1.14816623097032e-08	5.3006636356927e-07
Up in G2R	Luteolin	C01514	MP10795.pos	csin00941; csin00944; csin01110	Biosynthesis of secondary metabolites; Flavone and flavonol biosynthesis; Flavonoid biosynthesis	1.14816623097032e-08	5.3006636356927e-07
Up in G2R	Naringenin	C00509	MN11154.neg	csin00941; csin00943; csin01110	Biosynthesis of secondary metabolites; Flavonoid biosynthesis; Isoflavonoid biosynthesis	1.14816623097032e-08	5.3006636356927e-07
Up in G2R	Quercetin	C00389	MN12653.neg	csin00941; csin00944; csin01110	Biosynthesis of secondary metabolites; Flavone and flavonol biosynthesis; Flavonoid biosynthesis	1.14816623097032e-08	5.3006636356927e-07
Up in G2R	Coniferin	C00761	MP14572.pos	csin00940; csin01110	Biosynthesis of secondary metabolites; Phenylpropanoid biosynthesis	0.0011125403741246	0.006821313647403
Up in G2R	Sinapyl alcohol	C02325	MP6405.pos	csin00940; csin01110	Biosynthesis of secondary metabolites; Phenylpropanoid biosynthesis	0.0011125403741246	0.006821313647403
Up in G2R	trans-4-Coumaric acid	C00811	MP4968.pos	csin00940; csin01110	Biosynthesis of secondary metabolites; Phenylpropanoid biosynthesis	0.0011125403741246	0.006821313647403
Up in G2R	Umbelliferone	C09315	MP4861.pos	csin01110	Biosynthesis of secondary metabolites	0.0237172773895181	0.087951570319463
